# Hydrophobic Bile Salts Induce Pro-Fibrogenic Proliferation of Hepatic Stellate Cells through PI3K p110 Alpha Signaling

**DOI:** 10.3390/cells11152344

**Published:** 2022-07-29

**Authors:** Sebastian Zimny, Dennis Koob, Jingguo Li, Ralf Wimmer, Tobias Schiergens, Jutta Nagel, Florian Paul Reiter, Gerald Denk, Simon Hohenester

**Affiliations:** 1 Department of Medicine II, University Hospital, LMU Munich, Marchioninistr. 15, 81377 Munich, Germany; sebastian.zimny@med.uni-muenchen.de (S.Z.); dennis.koob@med.uni-muenchen.de (D.K.); jingguo.li@med.uni-muenchen.de (J.L.); ralf.wimmer@med.uni-muenchen.de (R.W.); jutta.nagel@med.uni-muenchen.de (J.N.); reiter_f@ukw.de (F.P.R.); gerald.denk@med.uni-muenchen.de (G.D.); 2 Department of General, Visceral and Transplantation Surgery, University Hospital, LMU Munich, Marchioninistr. 15, 81377 Munich, Germany; tobias.schiergens@med.uni-muenchen.de; 3 Division of Hepatology, Department of Medicine II, University Hospital Würzburg, Oberdürrbacher Str. 6, 97080 Würzburg, Germany

**Keywords:** cholestasis, HSC, myofibroblast, chenodeoxycholate, phosphatidyl-inositol-3-kinase p110 alpha, Alpelisib, liver fibrosis

## Abstract

Bile salts accumulating during cholestatic liver disease are believed to promote liver fibrosis. We have recently shown that chenodeoxycholate (CDC) induces expansion of hepatic stellate cells (HSCs) in vivo, thereby promoting liver fibrosis. Mechanisms underlying bile salt-induced fibrogenesis remain elusive. We aimed to characterize the effects of different bile salts on HSC biology and investigated underlying signaling pathways. Murine HSCs (mHSCs) were stimulated with hydrophilic and hydrophobic bile salts. Proliferation, cell mass, collagen deposition, and activation of signaling pathways were determined. Activation of the human HSC cell line LX 2 was assessed by quantification of α-smooth muscle actin (αSMA) expression. Phosphatidyl-inositol-3-kinase (PI3K)-dependent signaling was inhibited both pharmacologically and by siRNA. CDC, the most abundant bile salt accumulating in human cholestasis, but no other bile salt tested, induced Protein kinase B (PKB) phosphorylation and promoted HSC proliferation and subsequent collagen deposition. Pharmacological inhibition of the upstream target PI3K-inhibited activation of PKB and pro-fibrogenic proliferation of HSCs. The PI3K p110α-specific inhibitor Alpelisib and siRNA-mediated knockdown of p110α ameliorated pro-fibrogenic activation of mHSC and LX 2 cells, respectively. In summary, pro-fibrogenic signaling in mHSCs is selectively induced by CDC. PI3K p110α may be a potential therapeutic target for the inhibition of bile salt-induced fibrogenesis in cholestasis.

## 1. Introduction

Chronic cholestatic liver diseases encompass a broad spectrum of entities such as primary biliary cholangitis (PBC), primary sclerosing cholangitis (PSC), and congenital cholestatic syndromes such as progressive familial intrahepatic cholestasis (PFIC) [1]. Chronic cholestasis frequently leads to liver fibrosis and, eventually, end-stage liver disease with its subsequent complications. Today, cholestatic liver diseases are therefore among the leading reasons for liver transplantation [2].

Different pathogenetic pathways are involved in the different cholestatic disorders, yet ultimately the systemic and hepatic accumulation of hydrophobic bile salts is a common pathogenic feature [3,4,5]. In PBC, for example, systemic bile salt levels have been reported to be increased 20-fold in advanced stages [6]. Current pathophysiologic concepts of chronic cholestatic liver diseases therefore emphasize pro-fibrotic properties of accumulating hydrophobic bile salts. This widely accepted hypothesis was brought forward in the 1970s [4,7], and to this day, accumulation of hydrophobic bile salts is seen as a driving force of fibrosis in cholestatic liver disease [8,9,10,11]. However, as summarized recently [12], experimental evidence to support this hypothesis remains scarce. In established animal models of cholestasis, such as the Mdr2 knockout mouse or bile duct ligation (BDL), liver fibrosis may be caused by biliary damage rather than by the associated accumulation of bile salts. In models without biliary damage, such as the knockout mouse for the bile salt export pump (Bsep), bile salt accumulation, importantly, does not lead to liver fibrosis [13,14]. What might be the reason for the striking lack of liver fibrosis in murine hepatocellular cholestasis? One important species difference between mice and humans is the different bile salt pool, which is rather hydrophilic in mice but enriched with hydrophobic, toxic bile salts in humans [3,12]. We could recently show that, indeed, when introducing a humanized bile salt pool in mice, hepatocellular cholestasis led to an expansion of hepatic stellate cells (HSCs) and liver fibrosis in vivo. This study thereby provided, for the first time, in vivo evidence of liver fibrosis induced by cholestasis [12].

Various potential mediators of cholestatic liver fibrosis have been proposed, such as bile salt-induced hepatocellular apoptosis induced by hydrophobic bile salts [9,10,15,16] and bile salt-induced release of cytokines, such as TGF-β, by hepatocytes due to subtoxic concentrations of bile salts with subsequent activation of HSCs [17].

HSCs, upon activation, produce an extracellular matrix and are a major driving force for liver fibrosis [18]. Their potential direct activation by (hydrophobic) bile salts has been scarcely investigated, and molecular pathways mediating bile salt-induced effects in HSCs remain elusive.

Bile salts are signaling molecules with pleiotropic effects in various cell types. In hepatocytes and cholangiocytes, bile salt-induced activation of different signaling pathways is well established [19,20,21,22], including Phosphatidyl-inositol-3-kinse (PI3K) signaling [16,23]. Class I PI3Ks are heterodimers, which can be formed from different catalytic subunits, namely, p110α, p110β, p110γ, or p110δ [16]. When activated, PI3K catalyzes the conversion of phosphatidylinositol-4,5-bisphosphate (PIP_2_) to phosphatidylinositol-3,4,5-triphosphate (PIP_3_). PIP_3_ recruits further proteins like serin–threonine kinase Protein kinase B (PKB), which is activated by phosphorylation and is one of the main target molecules of PI3K [24]. PKB controls crucial cell functions such as cell growth and proliferation [25]. Pharmacological inhibitors of PI3K include Wortmannin and LY294002, which are considered pan-inhibitors for all isoforms [26,27]. Inhibitors specific to individual isoforms include the p110α inhibitor Alpelisib (also referred to as BYL719), a novel drug approved by the FDA for the treatment of breast cancer [28,29].

In summary, accumulating hydrophobic bile salts in chronic cholestatic disease have been proposed as drivers of liver fibrosis for decades. Only recently could this concept be confirmed in an in vivo mouse model. Underlying molecular pathways, however, remain elusive. Here, we investigated the specific effects of hydrophilic and hydrophobic bile salts on HSC activation, proliferation, and collagen deposition and sought to characterize underlying signaling pathways in search for a druggable target in cholestatic liver fibrosis.

## 2. Materials and Methods

### 2.1. Primary Hepatic Stellate Cells and LX-2 Cells

Primary murine HSCs were isolated from female FVB/N mice at the age of 12 to 24 weeks and purified by pronase–collagenase perfusion followed by density gradient centrifugation in 13.2% Nycodenz (Axis-Shield PoC, Oslo, Norway), as described previously [18,30]. The study protocol was approved by local authorities. Primary human HSCs were isolated from human liver tissues provided by the Biobank of the Department of General, Visceral, and Transplantation Surgery, LMU Klinikum Munich, Germany, under the administration of the Human Tissue and Cell Research Foundation (HTCR), allocation number 2016-28 (LMU Klinikum Munich, 11 January 2017) [31]. LX-2 cells were purchased from Merck-Millipore, Darmstadt, Germany.

### 2.2. Cell Culture

Cells were cultivated in Dulbecco’s Modified Eagle Medium (Sigma-Aldrich, Taufkirchen, Germany) containing 10% fetal bovine serum (FBS, PAN-Biotech, Aidenbach, Germany) for murine hepatic stellate cells (mHSCs) in accordance with Friedman et al. [18] and as described previously [32], and 2% FBS for LX-2 cells, respectively, as recommended by the providing company and as described previously [30,33]. Antibiotics and antimycotics (penicillin/streptomycin and actinomycin B, Sigma-Aldrich, Taufkirchen, Germany) were added to prevent contamination of cultivated cells. Cells were maintained at 37 °C in a humidified atmosphere with 5% CO_2_ and 21% O_2_. LX-2 cells were split with Accutase^®^ (Sigma-Aldrich, Taufkirchen, Germany) when an 80 to 90% confluent monolayer was formed. mHSCs were stimulated with chenodeoxycholate (CDC, Sigma-Aldrich, Taufkirchen, Germany), ursodeoxycholate (UDC, Calbiochem, San Diego, CA, USA), cholate (Sigma-Aldrich, Taufkirchen, Germany), and deoxycholate (DC, Sigma-Aldrich, Taufkirchen, Germany), 100 µM each, in the absence or presence of the PI3K inhibitors Wortmannin (0.1 µM; Sigma-Aldrich, Taufkirchen, Germany), LY294002 (5 µM; Cayman Chemical, Ann Arbor, MI, USA), and the catalytic subunit p110α specific inhibitor Alpelisib (up to 5 µM; MedChem Express, Monmouth Junction, NJ, USA) as well as the subunit p110γ specific inhibitors AS604850 (2.5 µM; Sigma-Aldrich, Taufkirchen, Germany) and AS605240 (0.1 µM; Sigma-Aldrich, Taufkirchen, Germany). LX-2 cells were incubated with TGF-β1 (10 ng/mL; PeproTech, London, UK) and CDC (20 µM; Sigma-Aldrich, Taufkirchen, Germany) in the absence or presence of PI3K inhibitors. The bile salt and inhibitor diluent dimethyl sulfoxide (DMSO) was used as a control.

### 2.3. siRNA Protein Suppression

The expression of the PI3K catalytic subunit p110α was suppressed using Accell siRNA (Catalog ID: A-003018-21-0020; Dharmacon, Lafayette, CO, USA) according to the manufacturer’s protocol with Accell siRNA Delivery Media (Catalog No: #B-005000). LX-2 cells were incubated with 0.5 µM siRNA for 48 h followed by incubation with TGF-β1 after 24 h of resting time for a further 24 h. Accell non-targeting pool siRNA (Catalog ID: D-001910-10-20) was used as a control.

### 2.4. LDH Assay

Cell cytotoxicity was evaluated by measuring lactate dehydrogenase (LDH) activity in the supernatants of cultured cells. Cell supernatant (500 µL) was mixed with phosphate buffer with pyruvate (500 µL, 0.6 mM) and NADH solution (10 µL, 10 mg/mL). Enzyme activity was determined photometrically with an Ultrospec 3100 pro UV/Visible Spectrophotometer (Biochrom, Cambridge, UK) at 339 nm wavelength. Then 0.1% Triton X-100 (Sigma-Aldrich, Taufkirchen, Germany) was added to cells 10 min prior to the LDH assay for evaluation of the maximal cytotoxic effect.

### 2.5. WST Assay

Cell viability was measured by the water-soluble tetrazolium (WST) assay (Roche, Mannheim, Germany), according to the manufacturer’s instructions, with the EASY READER SFplus (SLT-Labinstruments, Salzburg, Austria).

### 2.6. BrdU Assay

Hepatic stellate cell proliferation was quantified after 7 days in cell culture by the BrdU assay (Roche, Mannheim, Germany) after labeling for 24 h and antibody incubation for 90 min, according to the manufacturer’s instructions, with the EASY READER SFplus (SLT-Labinstruments, Salzburg, Austria).

### 2.7. DNA Quantification Assay

The total DNA amount in cells as a surrogate of cell number was determined after 14 days in cell culture by the PicoGreen^TM^ dsDNA assay (Invitrogen, Eugene, OR, USA) according to the manufacturer’s protocol, with a CytoFluor Multi-Well Plate Reader Series 4000 (PerSeptive Biosystems, Framingham, MA, USA).

### 2.8. Collagen Quantification In Vitro

Collagen quantification in vitro was performed as described previously [12]. In brief, cells were stained after 14 days of culture with 0.1% Sirius Red (Sigma-Aldrich, Taufkirchen, Germany) in saturated picric acid for 1 h. Adherent dye was dissolved in 50% methanol/sodium hydroxide (50 mM) after washing with ethanol. Absorption was photometrically measured at 540 nm with an Ultrospec 3100 pro UV/Visible Spectrophotometer (Biochrom, Cambridge, UK).

### 2.9. Cell Counting

Cell counting was performed as described previously [30]. mHSCs were fixed in 4% paraformaldehyde in phosphate-buffered saline (PBS; Sigma-Aldrich, Taufkirchen, Germany) and incubated with Hoechst 33342 (Fluka, Buchs, Switzerland) for 5 min. Microscopy was performed with a Zeiss Axiovert 135 TV microscope with a Zeiss AxioCam MRm camera (objective lens Fluar 10×/0.50), using Zeiss AxioVision SE64 Rel. 4.9 imaging software. Nuclei count was performed with ImageJ software (Version 1.53c, Wayne Rasband, National Institutes of Health, Bethesda, MD, USA).

### 2.10. Immunoblotting

Immunoblotting was performed as described previously [30]. Briefly, cells were lysed, protein concentrations of lysates were determined, and protein was loaded in equal amounts and separated by 10% sodium dodecyl sulphate polyacrylamide gel electrophoresis (SDS–PAGE). For PI3K p110 isoforms, 8% gels were used. Proteins were transferred to polyvinylidene difluoride (PVDF) membranes (Merck-Millipore, Darmstadt, Germany) on a semi-dry blot system (graphite electrodes produced in house by the technical staff of the Institute of Laboratory Medicine, University hospital, LMU Munich). Membranes were blocked in 1% casein (Carl Roth, Karlsruhe, Germany) for 30 min and incubated with monoclonal primary antibodies diluted in casein or albumin (Carl Roth, Karlsruhe, Germany) against α-smooth muscle actin (αSMA, 1:5000 for mHSCs and 1:1000 for LX-2 cells in 1% casein; Catalog ID: A2547, Sigma-Aldrich, Taufkirchen, Germany), PI3K p110α (1:1000 in 3% albumin; Catalog ID: #4249, Cell signaling, Danvers, MA, USA), PI3K p110β (1:1000 in 3% albumin; Catalog ID: #3011, Cell signaling, Danvers, MA, USA), PI3K p110γ (1:1000 in 3% albumin; Catalog ID: #5405, Cell signaling, Danvers, MA, USA), PI3K p110δ (1:1000 in 3% albumin; Catalog ID: #34050, Cell signaling, Danvers, MA, USA), PKB (1:1000 in 5% albumin; Catalog ID: #9272, Cell signaling, Danvers, MA, USA), Phospho PKB Ser473 (pPKB; 1:1000 in 5% albumin; Catalog ID: #4060, Cell signaling, Danvers, MA, USA), and glyceraldehyde 3-phosphate dehydrogenase (GAPDH; 1:10,000 for mHSCs and 1:50,000 for LX-2 cells in 1% casein; Catalog ID: ab8245, Abcam, Cambridge, UK) overnight at 4 °C followed by incubation with secondary goat anti-mouse IgG-HRP (1:10,000 in 1% casein; Catalog ID: #170-0616, Bio-Rad Laboratories, Feldkirchen, Germany) or goat anti-rabbit IgG-HRP (1:10,000 in 1% casein; Catalog ID: #172-1019, Bio-Rad Laboratories, Feldkirchen, Germany) as appropriate. Visualization was performed by ChemoCam (INTAS, Göttingen, Germany) after incubation with Clarity^TM^ Western ECL Substrate (Bio-Rad Laboratories, Germany). Densitometry was performed with TINA Image Analysis software version 2.10 g (Raytest, Straubenhardt, Germany).

### 2.11. Statistical Analysis

Statistical analysis and visual presentation of results were performed with GraphPad Prism (Version 8, GraphPad Software, Inc., San Diego, CA, USA), applying ANOVA with Fisher’s least significant difference (LSD) post hoc test, Student’s *t*-test, or Tukey HSD as appropriate. A probability value of <0.05 was considered statistically significant.

## 3. Results

### 3.1. The Hydrophobic Bile Salt CDC Specifically Promotes Proliferation and Expansion of HSCs with Subsequent Deposition of Collagen

**Figure 1:**
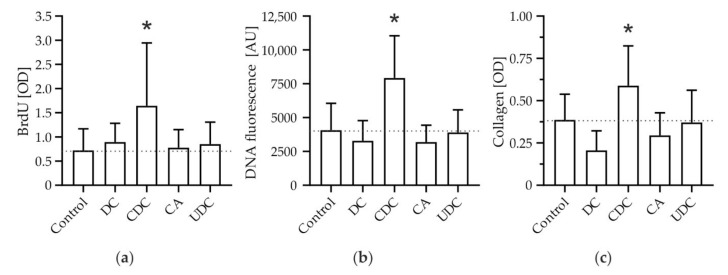
Chenodeoxycholate (CDC), but no other bile salt, specifically promotes hepatic stellate cell (HSC) expansion and extracellular matrix deposition. Primary murine HSCs were stimulated with deoxycholate (DC), CDC, cholate (CA), and ursodeoxycholate (UDC), respectively (100 µM, each). Controls were treated with diluent (0.01% dimethyl sulfoxide). (**a**) Proliferation was determined after 7 days by BrdU assays (*n* = 9). (**b**) The amount of DNA was used as a surrogate of cell number and quantified by PicoGreen^TM^ dsDNA assays after 14 days (*n* = 5). (**c**) Collagen deposition was determined by Sirius Red staining after 14 days (*n* = 9). Results are shown as mean ± SD (* *p* < 0.05 vs. control, Fisher’s LSD).

Isolated murine HSCs were incubated with the hydrophobic bile salts DC and CDC as well as the more hydrophilic bile salts CA and UDC (100 µM, each). Proliferation was determined by bromodeoxyuridine (BrdU) cell proliferation assays after 7 days, because at this timepoint, mHSCs showed the highest proliferation rate under non-stimulated conditions (Supplementary Figure S1). Incubation with CDC, the predominant bile salt accumulating in human cholestatic liver disease, but not with any other bile salt tested, resulted in an increase of mHSC proliferation (2.28-fold ± 1.80; Figure 1a). Next, we evaluated cell mass to confirm that increased proliferation translates into increased cell mass. Again, only incubation with CDC resulted in an increased amount of DNA mass, a surrogate of cell number, after long term culture (1.95-fold ± 0.77; Figure 1b). Most importantly, intra- and extracellular deposition of collagen, too, increased only upon stimulation with CDC (1.52-fold ± 0.61; Figure 1c).

In summary, the predominant bile salt accumulating in human cholestasis, CDC, but no other (hydrophilic or hydrophobic) bile salts, induced proliferation and expansion of cell mass and promoted collagen deposition by HSCs in vitro.

### 3.2. CDC-Induced Pro-Fibrotic Effects in HSC Engage PI3K-PKB Signaling

**Figure 2:**
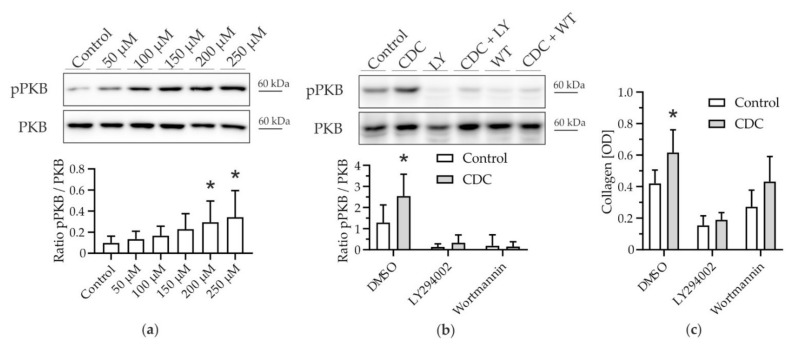
Phosphorylation of Protein kinase B (PKB) associates with CDC-induced HSC activation. Murine hepatic stellate cells (mHSCs) were stimulated with CDC in the presence or absence of Phosphatidyl-inositol-3-kinase (PI3K) pan-inhibitors. (**a**) Phosphorylation of PKB (pPKB) was detected after stimulation with CDC (50–250 µM, control 0.01% dimethyl sulfoxide) by Western blotting (*n* = 6). Results are shown as mean ± SD (* *p* <0.05 vs. control, Fisher’s LSD). (**b**) HSCs were stimulated with CDC (100 µM) for 4 h on day 7 in the absence or presence of PI3K inhibitors LY294002 (LY; 5 µM) or Wortmannin (WT; 0.1 µM), respectively. Phosphorylation of PKB was confirmed by Western blotting (*n* = 8–11). (**c**) Deposition of collagen was determined after 14 days of stimulation with CDC (100 µM) in the absence or presence of LY294002 (5 µM) and Wortmannin (0.1 µM), respectively, by Sirius Red staining (*n* = 3–5). Controls were treated with diluent (0.1% dimethyl sulfoxide). Results are shown as mean ± SD (* *p* < 0.05 vs. control, Student’s *t*-test).

Activation of PI3K has been established as an important bile salt-induced signaling pathway in hepatocytes [16]. Therefore, we explored the engagement of PI3K in HSCs using the phosphorylation of PKB as a downstream readout for PI3K activity. Stimulation of mHSCs with CDC resulted in phosphorylation of PKB in a dose-dependent manner (Figure 2a).

CDC-induced PKB phosphorylation (1.98-fold ± 0.8) was abolished by the PI3K pan-inhibitors LY294002 and Wortmannin (Figure 2b). When PI3K-PKB signaling was inhibited, CDC-induced collagen deposition was ameliorated (Figure 2c).

Thus, CDC specifically activated the PI3K/PKB signaling cascade and inhibition of this pathway blocked pro-fibrotic effects of CDC.

### 3.3. p110α Is the Catalytic PI3K Isoform Predominantly Mediating Pro-Fibrotic Effects of CDC

**Figure 3:**
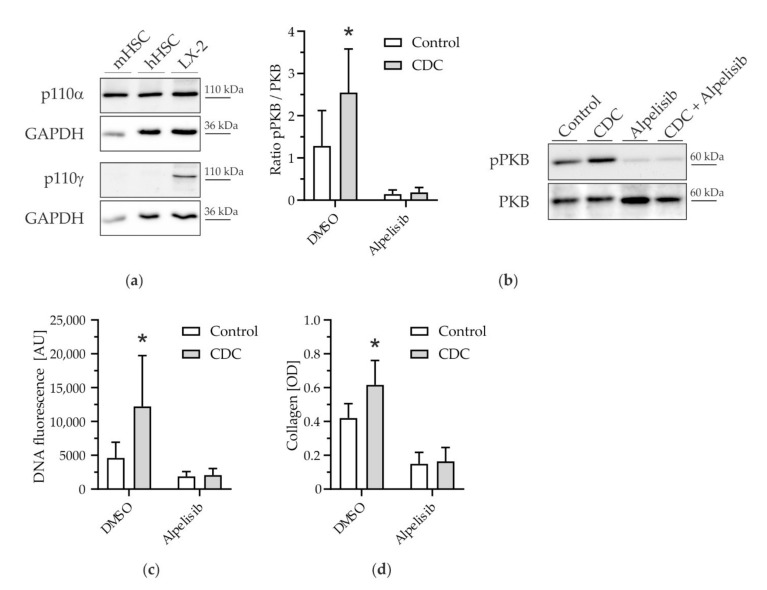
Pro-fibrogenic effects of CDC in HSCs are mediated by PI3K p110α. (**a**) Protein expression of catalytic PI3K isoforms p110α and p110γ in hepatic stellate cells (mHSCs: murine hepatic stellate cells; hHSCs: human hepatic stellate cells; LX 2: HSC cell line LX 2). GAPDH was used as a loading control. (**b**) mHSCs were stimulated with CDC (100 µM) in the presence or absence of the PI3K p110α inhibitor Alpelisib (5 µM) on day 7 of cell culture. PKB phosphorylation was determined by Western blotting after 4 h of stimulation (*n* = 4–8). (**c**) mHSCs were stimulated for 14 days with CDC (100 µM) in the presence or absence of Alpelisib (5 µM). The DNA amount was determined by DNA quantification assay (*n* = 10–12). (**d**) Collagen deposition in mHSCs was determined by Sirius Red staining after 14 days of culture after simulation with CDC (100 µM) in the presence or absence of Alpelisib (5 µM) (*n* = 5). Controls were treated with diluent (0.1% dimethyl sulfoxide). Results are shown as mean ± SD (* *p* < 0.05 vs. control, Student’s *t*-test).

As demonstrated previously in hepatocytes, different bile salts may selectively target different catalytic subunits of PI3K [16]. Hydrophobic bile salts were found to predominantly activate PI3K p110γ in hepatocytes to mediate their apoptotic effects. However, in mHSCs, the observed CDC-induced stimulation of HSCs was ameliorated by the pan-PI3K inhibitors LY294002 and Wortmannin (Figure 2c), but not by specific inhibition of PI3K p110γ (Supplementary Figure S2). Although LY294002 is widely referred to as a PI3K pan-inhibitor, its specificity for p110α is 10-fold higher than for p110γ [34]. Therefore, we determined protein expression of p110α and p110γ in HSCs. While p110α was present in both murine and human primary HSCs and in LX-2 cells (a human HSC-cell line), p110γ expression was absent in primary human and murine stellate cells (Figure 3a). Therefore, we further investigated the role of PI3K p110α in CDC-induced stimulation of HSCs. We chose a potent specific inhibitor of PI3K p110α, Alpelisib, a novel drug approved for the treatment of breast cancer [28,29]. CDC-induced activation of PKB (1.69-fold ± 0.69; Figure 3b), expansion of cell mass (2.64-fold ± 1.63; Figure 3c), and collagen deposition (1.47-fold ± 0.34; Figure 3d) were abolished by co-treatment with Alpelisib. Importantly, Alpelisib had no toxic effects on HSCs in the concentrations applied (Supplementary Figure S3).

In brief, inhibition of the p110α catalytic isoform of PI3K ameliorated pro-fibrogenic effects of CDC in HSCs.

### 3.4. Activation of the Human HSC Cell Line LX-2 Depends on PI3K p110α Signaling

Having demonstrated pro-fibrotic effects of CDC via PI3K p110α in murine HSCs, we aimed to translate our findings into the human setting by using the human HSC cell line LX-2. It is well established that LX-2 cells are activated and produce an extracellular matrix upon stimulation with TGF-β [35]. The production of αSMA by LX-2 cells upon treatment with TGF-β was confirmed by Western blotting (Supplementary Figure S4). Both the nonspecific PI3K inhibitors Wortmannin and LY294002 as well as Alpelisib reduced TGF-β1-induced expression of αSMA, i.e., activation of LX-2 cells (Supplementary Figure S4). None of the inhibitors used had any relevant cytotoxicity (Supplementary Figure S5).

Pharmacologic inhibitors are burdened with an innate risk of off-target effects. Therefore, to confirm our findings, we knocked down PI3K p110α in LX-2 cells by the use of siRNA. PI3K p110α protein expression was diminished following treatment with siRNA compared to non-targeting pool siRNA (Supplementary Figure S6). The expression of other PI3K p110-isoforms (p110β/p110γ/p110δ) in LX-2 cells remained unaltered after p110α siRNA treatment (Supplementary Figure S6). Following knockdown of PI3K p110α, TGF-β1-induced αSMA protein expression was diminished (Supplementary Figure S6).

**Figure 4:**
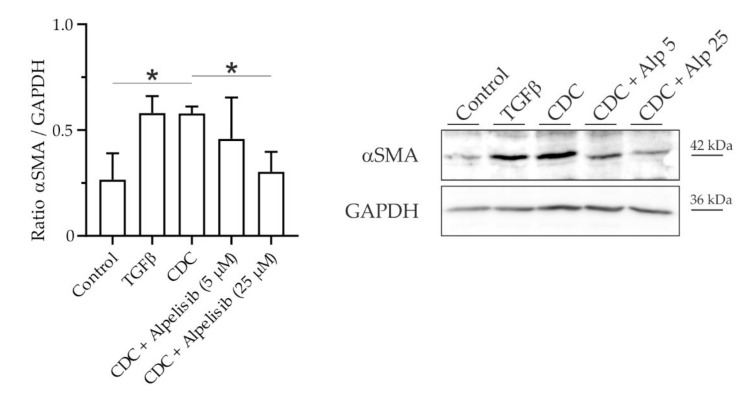
CDC-induced activation of the HSC cell line LX-2 is reduced by the PI3K p110α-specific inhibitor Alpelisib. The human HSC cell line LX-2 was incubated with CDC (20 µM) in the presence or absence of Alpelisib (Alp, 5 and 25 µM) for 24 h. The control was treated with diluent (0.1% dimethyl sulfoxide). TGF-β1 (10 ng/mL) served as a positive control for activation of LX-2 cells. αSMA protein expression was determined by Western blotting (*n* = 4) and normalized to GAPDH. Results are shown as mean ± SD (* *p* < 0.05 vs. control or co-incubation with Alpelisib 25 µM, Tukey HSD).

TGF-β is the classical stimulant to induce activation of LX-2 in vitro. For the purpose of our study, and extrapolating our findings in primary HSCs, we tested alternative activation of LX-2 by CDC. Indeed, stimulation of LX-2 with CDC led to the activation and αSMA expression to a similar degree as the positive control TGF-β1. Upon co-stimulation with Alpelisib, CDC-induced activation of LX-2 was ameliorated in a dose-dependent manner (Figure 4).

Thus, both inhibition and knockdown of PI3K p110α, respectively, were able to prevent activation of the human HSC cell line LX-2 by CDC and TGF-β, respectively.

## 4. Discussion

For decades, bile salts accumulating in chronic cholestatic liver diseases have been considered to be a driving force of liver fibrosis [4,7,8,9,10,11]. However, in vivo evidence to support this view has been lacking. Recently, in a model of hepatocellular cholestasis independent of inflammatory triggers or biliary damage, we could show that hydrophobic bile salts promote the expansion of HSCs in the liver [12]. The study suggested that the composition of bile salts was crucial for the pro-fibrogenic effect in vivo, since liver fibrosis only developed in the presence of a hydrophobic, humanized bile salt pool. To further unravel the pathophysiology of chronic cholestatic liver disease, we aimed to dissect the different effects of hydrophilic and hydrophobic bile salts on the biology of HSCs to investigate underlying signaling pathways and thereby find possibly amenable targets for therapy.

We could show in murine primary HSCs that CDC, but no other hydrophilic or hydrophobic bile salt, promoted proliferation of HSCs (Figure 1). This pro-proliferative effect was associated with an increase in cell mass and subsequent collagen deposition. CDC led to phosphorylation, i.e., activation of PKB in HSCs (Figure 2); conversely, upstream inhibition of PI3K prevented both PKB phosphorylation and CDC-induced collagen deposition, suggesting CDC-signaling through PI3K. By isoform expression analysis and the use of the p110α isoform-specific pharmacologic inhibitor Alpelisib, we identified PI3K p110α as a mediator of bile salt-induced pro-fibrogenic signaling (Figure 3). This finding was then translated into the human setting, as activation of the human HSC cell line LX2 by TGF-β1 could be inhibited by both pharmacological intervention with Alpelisib (Supplementary Figure S4) and siRNA-mediated knockdown of p110α (Supplementary Figure S6). We could further demonstrate direct activation of LX-2 cells by CDC (Figure 4). This activation was diminished by the PI3K p110α specific inhibitor Alpelisib. In summary, we provide evidence that human hydrophobic bile salts in particular are potent mediators of liver fibrosis. Furthermore, we identify PI3K p110α as a potential, druggable target in cholestatic liver disease.

TGF-β is a well-characterized molecule inducing HSC activation and involving Smad2/3 signaling [36,37]. Therefore, we investigated Smad2/3 signaling in CDC-induced HSC activation. However, a switch from C-terminally phosphorylated to linker phosphorylated Smad2/3 after short (up to 4 h) and long time (up to 14 days) incubation with CDC was not detectable, neither in murine hepatic stellate cells nor in LX-2 cells (data not shown).

Pro-proliferative effects of bile salts on HSCs (i.e., increase in BrdU incorporation) have only been demonstrated in two previous studies, and subsequent pro-fibrogenic down-stream effects (e.g., cell mass, collagen deposition) have never been investigated. Both hydrophilic and hydrophobic bile salts seemed to evoke pro-proliferative signaling in the first study [38]; however, our results are in line with the more recent work demonstrating a predominant effect of hydrophobic bile salts [39]. This difference is of particular importance when studying chronic cholestatic liver disease in vivo. While the bile salt pool in rodents is rather hydrophilic, the predominant bile salt accumulating in human cholestasis is the hydrophobic bile salt CDC (representing about 40% of the human bile salt pool [40]). As mentioned above, those previous reports were restricted to markers of enhanced proliferation, namely, BrdU assays [38,39]. Here, we characterized the bile salt-induced pro-fibrogenic expansion of HSC mass and subsequent collagen deposition. These readouts therefore emphasize the potential clinical importance of bile salts’ effects on HSCs [12].

Bile salts are well-established signaling molecules in various cell types and tissues. We have previously described a role for PI3K in bile salt-mediated signaling in hepatocytes [16,23,41]. Hence, we focused on this signaling pathway in HSCs and found the PI3K/PKB axis to be activated by stimulation with CDC. Conversely, inhibition of PI3K prevented CDC-induced pro-fibrogenic effects. Due to the pleiotropic effects of PI3K, however, pan-inhibitors of this signaling axis cannot be exploited therapeutically. We therefore sought to identify the specific catalytic subunit involved. In hepatocytes, p110γ has been shown to specifically transmit signaling by hydrophobic bile salts [16]. In primary HSCs, however, we found that this isoform is not expressed. This is in accordance with a study by Nakhaei-Rad et al., who showed negligible PI3K p110γ expression in HSCs derived from Wistar rats, and a further decrease during HSC activation [42]. This study also put into question the potential role of PI3K p110β, which according to The Human Protein Atlas [43] (Available online: http://www.proteinatlas.org, accessed on 1 June 2022) is marginally expressed in human stellate cells. However, we found the third-class IA catalytic isoform, PI3K p110α, to be expressed in both murine and human HSCs and could demonstrate its role in HSC activation by both pharmacologic intervention and specific knockdown. We used Alpelisib, a PI3K inhibitor that inhibits p110α about 50 times more potently than other isoforms [28]. In both murine and human HSCs, we could exclude cytotoxic effects in the concentrations used (Supplementary Figures S3 and S5). It is noteworthy that Alpelisib has been used in vitro, e.g., in pancreatic NET cell lines, in concentrations up to 250 µM [44].

Our results are in line with one previous study showing that adenovirus-transduced expression of dominant negative PI3K attenuated HSC activation [45], although this study did not further specify potential isoform-specific effects. The role of the catalytic subunit p110α has not been extensively studied, although its inhibition by compound HS-173 has been investigated in a model of liver fibrosis independent of cholestasis [46].

We are only beginning to unravel bile salt-induced signaling in HSCs. From experience, e.g., in hepatocytes, other signaling pathways may be of importance too, such as MEK-dependent signals, as suggested in our previous study [12].

In summary, our study adds new insights into the pathomechanism of hydrophobic bile salt-induced fibrosis in chronic cholestatic liver disease and highlights the role of the PI3K subunit p110α in HSC-mediated fibrogenesis. Thus, we could identify a promising, druggable target in the therapy of cholestatic liver disease. Given the very limited established pharmacological treatment options in chronic cholestasis and the resulting unmet clinical need, the newly identified PI3K p110α axis deserves further exploration.

## 5. Conclusions

The hydrophobic bile salt CDC induces pro-fibrogenic signaling and collagen deposition in murine HSCs, delineating a potential mechanism of liver fibrosis in chronic cholestasis. Bile salt-induced effects in HSC may be mediated, in part, by the PI3K catalytic subunit p110α. A role of PI3K p110α in fibrogenesis was reproduced in the TGF-β1- and CDC-induced activation of the human HSC cell line LX-2.

## Data Availability

The data presented in this study are available on request from the corresponding author. The data are not publicly available due to privacy reasons.

## Supplementary Materials

The following supporting information can be downloaded at: https://www.mdpi.com/article/10.3390/cells11152344/s1, Figure S1: Murine hepatic stellate cells (mHSC) show maximum proliferative activity on day 7 of cell culture; Figure S2: Chenodeoxycholate (CDC)-induced increase in HSC mass is unaffected by PI3K p110g inhibition; Figure S3: The PI3K p110a inhibitor Alpelisib does not exhibit toxic effects on mHSC; Figure S4: TGF-b1-induced activation of the HSC cell line LX-2 is reduced by PI3K inhibitors; Figure S5: PI3K inhibitors have negligible cytotoxic effects on the human HSC cell line LX-2; Figure S6: siRNA-knockdown of PI3K p110a impedes TGF-b1-induced activation of the human HSC cell line LX-2.
