# Supplementary material for: Hydrophobic Bile Salts Induce Pro-Fibrogenic Proliferation of Hepatic Stellate Cells through PI3K p110 Alpha Signaling
Source: Cells. 2022 Jul 29;11(15):2344. doi: 10.3390/cells11152344 (PMC9367387; doi:10.3390/cells11152344)
Supplement: Supplementary file 1 [file cells-11-02344-s001.zip › cells-1794485-supplementary.pdf]

cells

MDPI

Supplementary Materials

Hydrophobic Bile Salts Induce Pro-Fibrogenic Proliferation of Hepatic Stellate Cells through PI3K p110 Alpha Signaling

Sebastian Zimny [1], Dennis Koob [1], Jingguo Li [1], Ralf Wimmer [1], Tobias Schiergens [2], Jutta Nagel [1], Florian Paul Reiter [1,3], Gerald Denk [1] and Simon Hohenester [1,*]

[1] Department of Medicine II, University Hospital, LMU Munich, Marchioninistr. 15, 81377 Munich, Germany; sebastian.zimny@med.uni-muenchen.de (S.Z.); dennis.koob@med.uni-muenchen.de (D.K.); jingguo.li@med.uni-muenchen.de (J.L.); ralf.wimmer@med.uni-muenchen.de (R.W.); jutta.nagel@med.uni-muenchen.de (J.N.); reiter_f@ukw.de (F.P.R.); gerald.denk@med.uni-muenchen.de (G.D.)

[2] Department of General, Visceral and Transplantation Surgery, University Hospital, LMU Munich, Marchioninistr. 15, 81377 Munich, Germany; tobias.schiergens@med.uni-muenchen.de

[3] Division of Hepatology, Department of Medicine II, University Hospital Würzburg, Oberdürrbacher Str. 6, 97080 Würzburg, Germany

* Correspondence: simon.hohenester@med.uni-muenchen.de; Tel.: +49-(0)89-4400-0

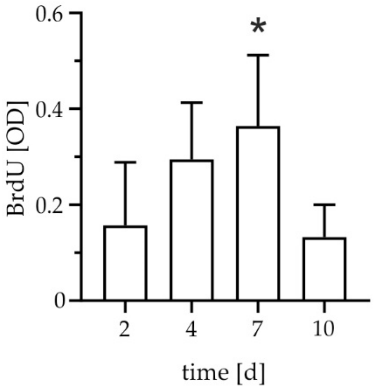

Figure S1. Murine hepatic stellate cells (mHSC) show maximum proliferative activity on day 7 of cell culture. mHSC were cultured on plastic culture dishes. Spontaneous proliferation was determined by BrdU assays on day 2, 4, 7 and 10 of cell culture (n = 5 – 8). Results are shown as mean ± SD (*p <0.05 vs. day 2, Tukey HSD).

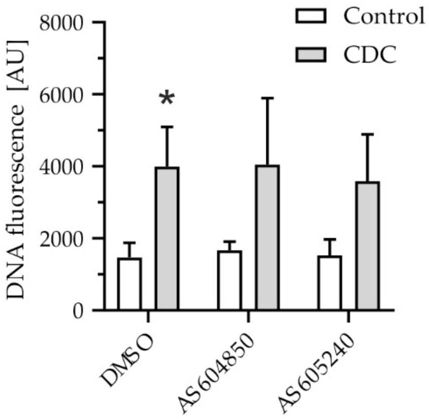

Figure S2. Chenodeoxycholate (CDC)-induced increase in HSC mass is unaffected by PI3K p110γ inhibition. mHSC were stimulated with CDC (250 µM). DNA amount was quantified by PicoGreen™ dsDNA assays after 14 days of culture. To suppress Phosphatidyl-inositol-3-kinase

(PI3K) p110γ signaling, specific pharmacologic inhibitors AS604850 (2.5 μM) and AS605240 (0.1 μM) were used. Dimethyl sulfoxide (0.1%) was used as control. Results are shown as mean ± SD (n = 5, *p<0.05 vs. control, Fisher's LSD).

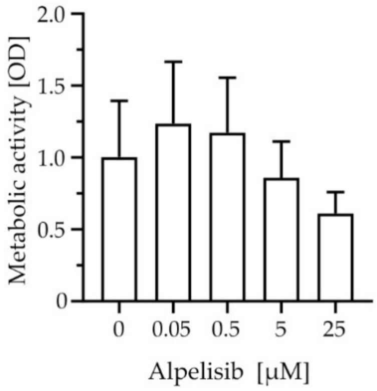

Figure S3. The PI3K p110α inhibitor Alpelisib does not exhibit toxic effects on mHSC. mHSC were treated with Alpelisib (0.05 to 25 μM) for 7 days. Cell viability was determined by WST assay (n = 6). A tendency is seen towards lower viability only in high concentrations (25 μM).

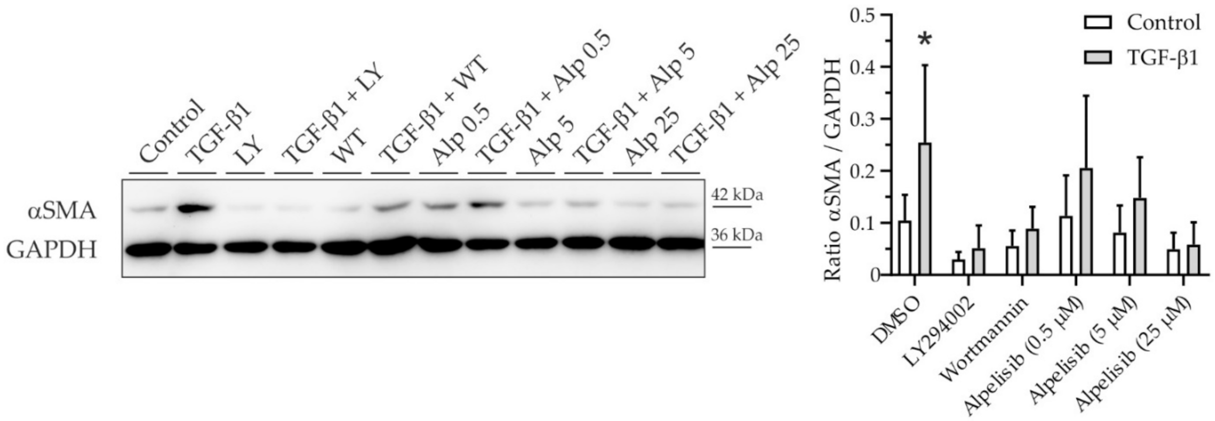

(**a**) (**b**)

Figure S4. TGF-β1-induced activation of the HSC cell line LX-2 is reduced by PI3K inhibitors. The human HSC cell line LX-2 was incubated with TGF-β1 (10 ng/ml) in presence or absence of PI3K inhibitors LY294002 (LY, 5 μM) and Wortmannin (WT, 0.1 μM) as well as the PI3K p110α specific inhibitor Alpelisib (Alp, 0.5, 5 and 25 μM) for 24 hours. Control was treated with diluent (0.1% dimethyl sulfoxide). (**a**, **b**) αSMA protein expression was determined by western blotting (n = 4 – 8) and normalized to GAPDH. Results are shown as mean ± SD (*p <0.05 vs. control, Fisher's LSD).

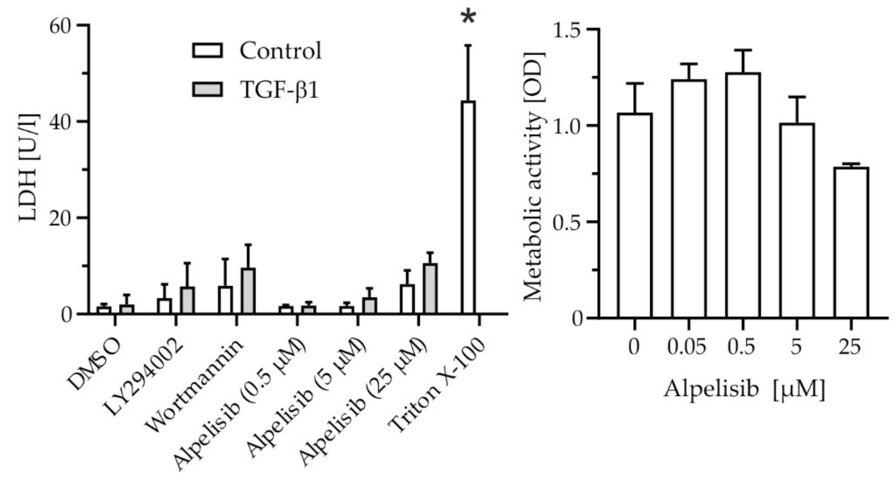

(a) (b)

Figure S5. PI3K inhibitors have negligible cytotoxic effects on the human HSC cell line LX-2. LX-2 cells were stimulated with PI3K pan-inhibitors LY294002 (5 μM) and Wortmannin (0.1 μM) as well as the PI3K p110α specific inhibitor Alpelisib (0.05 to 25 μM) in presence or absence of TGF-β1 (10 ng/ml) for 24 hours. 0.1% dimethyl sulfoxide was used as control. (**a**) Cytotoxicity was determined by LDH activity assay in cell culture medium (n = 5). Triton X-100 was used as positive control. (**b**) Cell viability was determined by WST assay (n = 3). A tendency is seen towards lower viability only in high concentrations of Alpelisib (25 μM). Results are shown as mean ± SD (*p <0.05 vs. control, Tukey HSD).

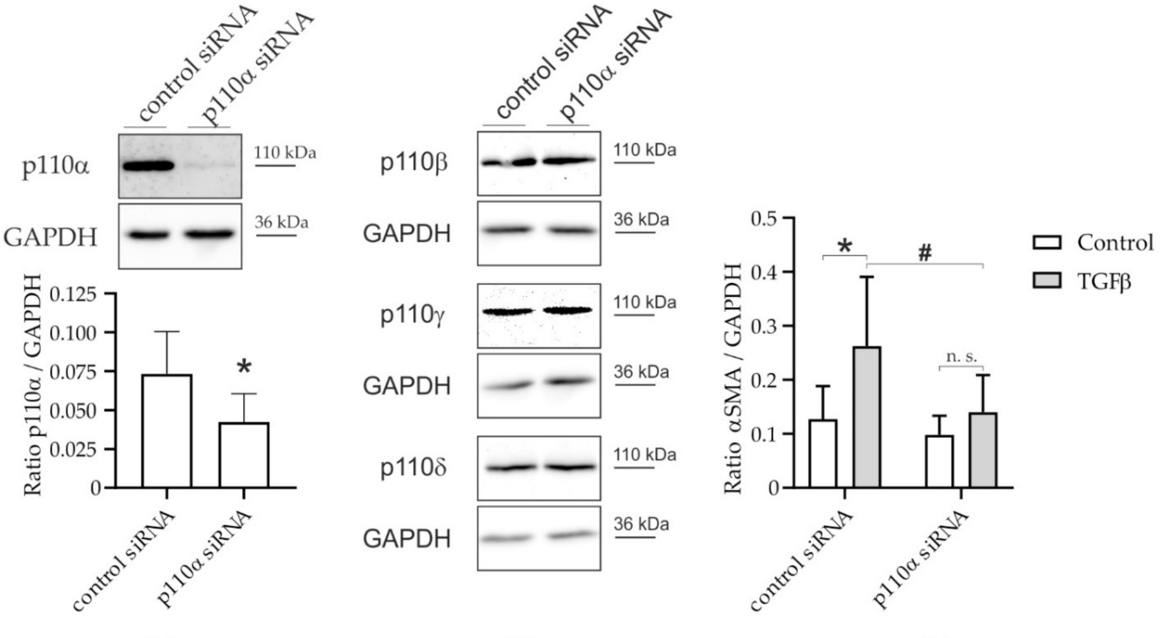

(a) (b) (c)

Figure S6. siRNA-knockdown of PI3K p110α impedes TGF-β1-induced activation of the human HSC cell line LX-2. LX-2 cells were incubated with siRNA targeted against PI3K p110α or non-targeting pool siRNA (0.5 μM) for 48 hours, followed by 24 hours of resting time and subsequent incubation with TGF-β1 for another 24 hours. (**a**) PI3K p110α protein expression in LX-2 cells was determined by western blotting. PI3K p110a protein expression was reduced by 42% (mean ± SD: control siRNA 0.073±0.027, p110α siRNA 0.042±0.018) after incubation with p110α siRNA (n = 8, *p <0.05 vs. control siRNA, Student's t-test). (**b**) Expression of PI3K isoforms p110β, p110γ and p110δ in LX-2 cells was not impaired by p110α siRNA incubation. (**c**) αSMA protein expression in LX 2 cells after stimulation with TGF-β1 was determined by western blotting (n = 8). Controls were treated with diluent (0.1% dimethyl sulfoxide). GAPDH was used as housekeeping protein. Results are shown as mean ± SD (*p <0.05 vs. control, #p <0.05 vs. p110α siRNA, Fisher's LSD).
